# Notes and News

**Published:** 1890-08-09

**Authors:** 


### Notes and News.

Collected and Communicated.

Tewkesbury Rural Hospital reports 161 in, and 775 out-patients for last year. The average cost of each in-patient was £2 13s. 5d. The proposed new children's ward is not yet commenced, but subscriptions are coming in rapidly, and the committee hope soon to start the bricklayers to work. The new matron, Miss Carroll, gives great satisfaction.

The ninety-first annual report of Sunderland Infirmary tells of 1,873 in-patients and 2,709 out-patients, treated at a cost per occupied bed for the year of £47 5s. 9d. Miss Forster has offered to build an isolation ward, which offer has been gratefully accepted by the committee. The president, Mr. Laing, is very anxious for a scheme of amalgamation between the kindred institutions of the town.

The man Wickens, who was found some three weeks ago near Eastbourne, with three nails driven into his head, has been charged with attempting to commit suicide. Dr. McQueen produced four nails which he had with difficulty withdrawn from the head of Wickens. These nails had penetrated three inches and gone into the brain, but to the surprise of the medical staff at the Memorial Hospital, Wickens had fully recovered. Wickens said he drove the nails into his head in succession with a hammer, and he had felt better in his head since the occurrence. This is a form of treatment better applied by the patient than the doctor. It is said that Wickens is now sane, but we rather dread his figuring in the papers again.

West Bromwich District Hospital treated 595 in, and 5,563 out-patients last year, at a total cost of £2,769. There is a balance due to the treasurer of nearly £600. The eye department has been working well for five months. The board have decided to erect on the land purchased in Lodge Road two circular wards adjoining, to contain one bed each; to remove the mortuary, laundry, &c., and to put up an operating room; to convert the present operating room into a ward with two beds; to build additional sleeping rooms for nurses; and to make sundry minor alterations and improvements.

It is rumoured that platform hypnotism is to be made the subject of a Bill before Parliament, introduced by the medical faculty, and designed to prohibit public exhibitions of hypnotism, on the ground that they increase insanity. From the article in the *Fortnightly Review* on "The Latest Discoveries in Hypnotism," we imagine it is high time this serious subject should no longer be used as a plaything by the ignorant.

The Royal Sea-Bathing Infirmary for Scrofula, at Margate, celebrates its centenary next July, and a great effort is to be made to collect £5,000 to set this unique and valuable institution upon a sounder financial basis. A Centenary Committee has been formed, and we wish them all success. During July Margate is always crowded, and the institution invites and repays inspection; anyone thoroughly understanding its good work must desire to lend it a helping hand.

Mr. Cursham Corner has written a little pamphlet on "The Feeding, Clothing, and General Care of Infants," which we heartily recommend to workers in the cause of family hygiene. The directions are given very clearly, and in the most simple language, so that even the most ignorant mother must comprehend the truths about the little ones there set forth. The pamphlet is a capital one for distribution by district visitors, and can be had from Reynolds, 23, Stepney Green, E.

Melbourne is much exercised as to how it shall elect the medical officers of its hospitals. At present the general body of subscribers to Melbourne Hospital elect the staff, and, of course, medical or surgical knowledge counts less than affable manners and personal influence. One lady subscriber was heard to remark she shouldn't vote for Mr. Jones, he was only a F.R.C.S.; she should vote for Mr. Smith, who was L.K.Q.C.P.I. Of course no one could be found to vote for a simple M.D. The chairman of the hospital has the audacity to say in the *Argus* that it is usual in London to elect the junior medical staff by subscribers' votes, and then promote them in rotation. We are afraid that chairman doesn't read The Hospital—a heinous sin.

At the meeting of the Hospital Sunday Fund, Sir Sydney Waterlow moved that it be referred to the Distribution Committee to confer with the Lord Mayor, with the view of calling a meeting of the managers of the leading hospitals to consider the possibility of arranging for some uniform system of accounts, and he believed it would be possible, by the proposed conference, to arrange for a systematic and uniform basis of accounts by which the public might judge of the financial position of the hospitals. The motion was carried. This is an important point; also the fact that the fund increases yearly, and has obviously the complete confidence of the public. Its duties, therefore, and its powers are likely to increase.

The Medical Scientific Exhibition connected with the tenth International Medical Congress at Berlin was opened on Saturday, in the Central Hall of the International Exhibition Buildings, a large number of German and foreign delegates to the Congress being present. Dr. Lassar, Secretary-General of the Congress, delivered the opening speech welcoming the members, and was followed by Professor Virchow, who expressed the thanks of the Congress to the Minister of Education, to the Senate of the Academy of Arts, and to the exhibitors for their endeavours to make the exhibition a success. Dr. Koehler, Director of the Imperial Board of Health, welcomed the assembly in the name of the Government. The Congress had been hard at work on subjects chiefly of popular interest, and all Europe and America has been following the proceedings with interest.

The following vacancies are announced this week, the amount of the salary in each case being given after the name of the institution:—

**Resident Medical Officers.**

Manchester Eye Hospital, House Surgeon—£70, board, etc.

Cheadle Convalescent Hospital, Resident Medical Officer—£150, board, etc.

Ripon Cottage Hospital, House Surgeon and Dispenser—£60, board, etc.

Torbay Hospital, House Surgeon and Dispenser—£80, board, etc.

Victoria Park Chest Hospital, House Physician—Board, etc.

Dudley Dispensary, Resident Medical Officer—£130, house, etc.

**Non-Resident Medical Officers.**

Gloucester Friendly Societies, Assistant Medical Officer—£120.

Counties of Ross and Cromarty, Medical Officer of Health—£120.

National Orthopædic Hospital, Hon. Assistant Surgeon, Surgical Registrar and Hon. Anæsthetist.

Camberwell Provident Dispensary, Medical Officer.

Wantage Union, Medical Officer—£40.

Manchester Consumption Hospital—Hon. Assistant Physician.

---

Bristol has been much agitated lately on medical matters, particularly by the rule of the General Hospital, which excludes from the medical staff candidates from Scotch or Irish schools. This is a question in which Lord Cadogan, Lord Spencer, and other members of the Select Committee are specially interesting themselves, and if Bristol has aught to say in defence of its policy of exclusiveness it had better say it at once. Considering what men the Edinburgh schools have produced, it does seem rather bumptious for little Bristol to refuse Scotch graduates a recognition.

---

The North of England Children's Sanatorium at Southport has this summer been augmented by a new wing, which was opened by the Mayoress in June. The dining-room has been enlarged by 28 feet 5 inches by 17 feet 8 inches; overhead a new room containing five beds has been erected for the nursing staff; and still higher an entirely new ward, in which are placed ten beds, has been built. This latter ward, which increases the inmates' sleeping accommodation from 70 to 80, has been appropriately named "The Harvey Ward," after the late Dr. Harvey. It is a particularly pleasant and cheerful room, well ventilated, and is to a great extent furnished, through the well-directed efforts of Miss Smith, one of the committee, who collected about £50 for that purpose. Over the fireplace hangs an excellent portrait of the late Dr. Harvey. The entire cost of the wing has been about £500, and, notwithstanding the largeness of this sum, it was opened entirely free from debt. The architects were Messrs. Mellor and Sutton, London Street. We are sorry this news is so late, and apologize to our kind correspondent who sent it for overlooking her letter so long.

---

"What fools men be" is specially proved when the public takes to doctoring its own ailments. Here comes to us an anxious letter from a distracted correspondent:—"I have took over 2 lbs. tomatoes daily for the last fortnight as a cure for dyspepsia. Can there be any truth in the enclosed?" The "enclosed" is a cutting from one of the evening papers, and runs as follows:—"Tomatoes and Cancer.—Like a correspondent, I have been a victim to dyspepsia, and, like him, have found tomatoes a cure; but when I told my doctor, he asked me if I was aware I was curing one evil to bring on a much more terrible one? He told me he, with other medical men, were of opinion that the terrible increase in cancer in this country was due to the increased consumption of tomatoes. I heard of a gentleman suffering from cancer who went to a specialist in Paris. The first question he asked him was, 'Have you been eating tomatoes?' Can any of your readers tell me if there is any truth in this?"—Yes, Sir or Madam, there is a great truth in this; the old old one, that the man who is his own doctor has a fool for his physician. If you suffer from cancer or dyspepsia it is wiser to consult a medical man than an evening paper.

Already a prompt and generous response has been made to our appeal for parcels for the adults in hospital wards at Christmas. Several promises of garments have been received, and one parcel has actually arrived. We should be obliged if friends would not forward their gifts to us yet; space is so precious in London, and our offices are none too large. Still it is delightful to think that holiday-makers both far and near are using their fingers in the cause of a cheerful Christmas in the adult wards of our large London hospitals. Such thoughtfulness and unselfishness is very encouraging, and we send our grateful thanks to those who have so promptly taken up our suggestion. Once more we repeat that we hope to receive from our readers in December a plentiful stock of shawls, knitted waistcoats, mittens, scarves, aprons, petticoats, crossovers, &c., for distribution amongst the hospitals.

---

Mr. Sidney Quennell, secretary to the Middlesex Hospital, writes a fervid plea for the arrest of bands in processions passing the hospital on Sundays. He says:—"Several of the patients were very much disturbed; our night nurses, who retire to rest at noon, were twice awakened, and many of them did not obtain more than two hours' sleep before having to go again on duty. My committee feels sure that if those who organise these processions only knew how much we suffer from them they would at least take care that the bands refrained from playing while passing the hospital; but processions occur so frequently, and the bodies which organise them appear to be so numerous, that probably the only way of reaching them is through the medium of the Press." This is an appeal to the people which we are sure will not remain unheeded.

---

In the interesting autobiography of Joseph Jefferson, in the *Century Magazine*, there is a pathetically humorous story of Artemus Ward: "Just before his death Robertson poured out some medicine in a glass, and offered it to his friend. Ward said, 'My dear Tom, I can't take that dreadful stuff.' 'Come, come,' said Robertson, urging him to swallow the nauseous drug, 'there's a dear fellow. Do now, for my sake. You know I would do anything for you.' 'Would you?' said Ward, feebly stretching out his hand to grasp his friend's, perhaps for the last time. 'I would, indeed,' said Robertson. 'Then you take it,' said Ward." This reminds us of a story told of Hood when he was dying; it was a stormy night, and one of the windows would rattle; the dying man glanced at it, and then at his wife. "It is the wind, dear," she said. "Put a peppermint lozenge on the sill," whispered the humourist, unable to resist a joke with his last breath.

---

Sheffield Public Hospital held its general meeting on July 30th. The plans for the new building were on the table. Mr. G. F. Lockwood read the annual report and statement of accounts, in which reference was made to the growth of the annual income of the institution, the amount received from subscriptions being £102 9s. 7d. more than last year, which, with other items, brought up the total income to £4,522 19s. 8d. The expenditure had been £113 15s. 4d. less than the previous year. The board still regretted that they had to point out a deficiency on the income account of £367 12s. 7d., but the legacies, amounting to £397 19s., covered the deficiency. During the past year a most valuable report upon the insufficiency and inefficiency of the present hospital building had been presented by the medical staff, and it most conclusively proved what had been so often mentioned in previous annual reports, how absolute was the necessity for a new building. Since the last report special donations to the amount of £588 had been received for this purpose, which amount had been invested until it would be required. The building fund now amounted to £1,347 12s. 4d.

